# Interactive Effects of Cabbage Aphid and Caterpillar Herbivory on Transcription of Plant Genes Associated with Phytohormonal Signalling in Wild Cabbage

**DOI:** 10.1007/s10886-016-0738-3

**Published:** 2016-08-16

**Authors:** Yehua Li, Marcel Dicke, Anneke Kroes, Wen Liu, Rieta Gols

**Affiliations:** Laboratory of Entomology, Wageningen University, P.O. Box 16, 6700 AA, Wageningen, The Netherlands

**Keywords:** Aphid infestation, Caterpillar infestation, Gene transcription, Genotypic variation, Plant defense, SA-JA antagonism

## Abstract

Plants are commonly attacked by a variety of insect herbivores and have developed specific defenses against different types of attackers. At the molecular level, herbivore-specific signalling pathways are activated by plants in response to attackers with different feeding strategies. Feeding by leaf-chewing herbivores predominantly activates jasmonic acid (JA)-regulated defenses, whereas feeding by phloem-sucking herbivores generally activates salicylic acid (SA)-regulated defenses. When challenged sequentially by both phloem-sucking and leaf-chewing herbivores, SA-JA antagonism may constrain the plant’s ability to timely and adequately divert defense to the second herbivore that requires activation of a different defensive pathway. We investigated the effect of the temporal sequence of infestation by the aphid *Brevicoryne brassicae* and three caterpillar species, *Plutella xylostella*, *Pieris brassicae*, and *Mamestra brassicae,* on the interaction between JA and SA signal-transduction pathways in three wild cabbage populations. We found no support for SA-JA antagonism, irrespective of the temporal sequence of herbivore introduction or the identity of the caterpillar species based on the transcript levels of the JA- and SA-regulated marker genes *LOX* and *PR-1,* respectively, at the examined time points, 6, 24, and 48 h. In general, infestation with aphids alone had little effect on the transcript levels of the two marker genes, whereas the three caterpillar species upregulated not only *LOX* but also *PR-1*. Transcriptional changes were different for plants from the three different natural cabbage populations.

## Introduction

Plants live in a hostile environment and are challenged by a diverse range of attackers, including microbial pathogens and insect herbivores that may attack the plant either simultaneously or sequentially. To cope with the diversity of biotic threats that may reduce the survival and fitness of plants, they are equipped with traits that prevent or reduce attack by biotic agents. These traits, both physical and chemical, can be constitutively expressed or may be activated or enhanced upon attack (Agrawal 1999; Dicke and Baldwin 2010; Karban and Baldwin 1997). To respond adequately to biotic threats, plants need to detect and differentiate between different attacker species. Following the perception and recognition of the attacking herbivore, plants activate an herbivore-specific signal-transduction network that leads to biochemical and physiological changes (De Vos et al. 2005; Erb et al. 2012; Wu and Baldwin 2009, 2010).

Specificity in the response to attackers allows plants to mount a defense that can more effectively cope with herbivore species with distinct life styles and feeding strategies (Howe and Jander 2008; Pieterse et al. 2009). Leaf chewing and phloem feeding insect herbivores are particularly well studied in relation to defense induction in plants. Leaf chewers remove plant tissues and can cause severe damage to plants, whereas piercing-sucking phloem-feeding herbivores feed more subtly, causing only minimal physical damage to plant tissues (Schoonhoven et al. 2005). At the molecular level, defenses against leaf-chewing and phloem-feeding herbivores are regulated by two major signal-transduction pathways controlled by the phytohormones jasmonic acid (JA), and salicylic acid (SA). Chewing herbivores generally activate defense responses regulated by JA, whereas piercing-sucking herbivores activate defense responses regulated by SA, although this generalization may oversimplify the complex interplay between hormone-regulated induced defenses (Howe and Jander 2008; Kessler and Baldwin 2002; Mewis et al. 2005; Thaler et al. 2012).

When plants are challenged by multiple herbivore species, crosstalk between defense-related phytohormonal signalling pathways may occur, which can help plants to fine-tune their response timely and plastically to the attackers encountered (Howe and Jander 2008; Pieterse et al. 2009; Stam et al. 2014). The best studied interaction between phytohormonal signalling pathways is the antagonistic interaction between JA- and SA-mediated signalling. The activation of the JA signalling pathway may interfere with the SA signalling pathway and *vice versa* when challenged simultaneously by leaf-chewing and phloem-feeding herbivores (Koornneef and Pieterse 2008; Pieterse et al. 2012; Thaler et al. 2012). The ecological consequence of this negative SA-JA crosstalk includes e.g., the enhanced performance of caterpillars and their parasitoids on aphid-infested plants as a result of the interference of SA signalling with JA-induced plant defenses (Li et al. 2014; Rodriguez-Saona et al. 2010; Soler et al. 2012). The negative interaction between JA- and SA-mediated signalling suggests that plants are constrained in their ability to cope with attack by multiple herbivore species that induce different signalling pathways.

The temporal sequence of herbivory may determine the outcome of the SA-JA crosstalk (Mouttet et al. 2013). For instance, a study on *Nicotiana attenuata* showed that the order of attack by phloem-feeding mirids and leaf-chewing tobacco hornworms is an important determinant explaining the differences in plant transcriptional responses (Voelckel and Baldwin 2004). Moreover, the leaf chewing *Spodoptera frugiperda* negatively affected the colonization of maize plants by the root feeder *Diabrotica virgifera*, but only when the leaf herbivore arrived earlier than the root herbivore (Erb et al. 2011). Thus, when attacked by different species sequentially, the kinetics of the plant’s response to the first attacker may limit the ability of the plant to divert its response to a second attacker that activates a different signal-transduction pathway. However, in Lima bean plants, the order of attack by JA-activating spider mites and SA-activating whiteflies (*Bemisia tabaci*) did not exhibit major effects on induced plant responses (Zhang et al. 2009). Similarly, the performance of *B. tabaci* was not affected by pre-infestation by a chewing leaf miner (*Tuta absoluta*) in tomato (Mouttet et al. 2013). However, in the same study system, pre-infestation with either whiteflies or a fungal biotrophic pathogen (*Oidium neolycopersici*), which also is associated with SA-regulated defense induction, negatively impacted the development of the leaf miner. These effects were either only locally expressed in the induced leaf (response to whiteflies) or both locally and systemic (response to pathogen) (Mouttet et al. 2013). These examples also suggest that the outcome of plant-mediated species interactions can be highly context-specific.

Although herbivores of the same feeding guild generally trigger the same major signalling pathway (Erb et al. 2012), plant responses to species of the same feeding guild are not exactly the same (Bidart-Bouzat and Kliebenstein 2011), which likely is the result of modulation of defense responses due to crosstalk at the molecular level (De Vos et al. 2005). For example, feeding by various lepidopteran species resulted in differential induction of the three major phytohormones involved in induced plant responses, as well as differential transcriptional responses (Diezel et al. 2009; Poelman et al. 2011; Zhu et al. 2015).

Closely related plant species may vary in their responses to the same type of herbivory (Schmidt et al. 2005). Moreover, within one species, heritable variation in resistance traits is an important component in the adaptation of plants to environmental stresses (Gols et al. 2008; Newton et al. 2009a; Wu and Baldwin 2010). Intraspecific variation was found for plant secondary metabolites, such as glucosinolates in brassicaceous plant species, in *Arabidopsis thaliana* accessions and wild cabbage, *Brassica oleracea*, populations ( Gols et al. 2008; Kliebenstein et al. 2001; Newton et al. 2009b) and crosstalk between SA- and JA-regulated defenses differed among *A. thaliana* accessions (Pieterse and Dicke 2007; Pieterse et al. 2009; Traw et al. 2003). In two accessions of *N. attenuata,* large differences in herbivory-induced early signalling events, such as MAPK activity, JA and ethylene production, and transcript accumulation of genes that encode transcription factors were recorded (Wu et al. 2008). Therefore, the underlying regulatory mechanisms of plant defense may vary among plant genotypes and populations.

The aim of this study was to investigate whether aphid- and caterpillar-induced plant responses interfere with each other through negative SA-JA crosstalk in different populations of wild cabbage. Underlying mechanisms that explain plant responses to herbivory rely to a large extent on studies performed on *A. thaliana* (De Vos et al. 2005; Koornneef and Pieterse 2008; Kroes et al. 2015; Pieterse et al. 2009). The question is to what extent the results of these studies are representative for plant responses to herbivory in general or for brassicaceous plants more specifically, as the interaction of Arabidopsis with herbivores in nature is limited due to their short life cycle early in the growing season (Harvey et al. 2007).

In this study, we used plants grown from seeds that originated from three wild cabbage populations that are known to differ in secondary plant chemistry (Gols et al. 2008; Harvey et al. 2011; Newton et al. 2009a), and interact in nature with the herbivores used in this study, the aphid *Brevicoryne brassicae* L. (Hemiptera, Aphididae), and three chewing lepidopteran species, caterpillars of *Plutella xylostella* (L.) (Plutellidae), *Pieris brassicae* L. (Pieridae), and *Mamestra brassicae* L. (Noctuidae), respectively (Newton et al. 2009b). We addressed the following questions: 1) What is the effect of the sequence of herbivore attack on SA-JA crosstalk? 2) How general is this response when using different species of chewing herbivores? and 3) Is there intra-specific variation in the plant’s responses to herbivory by aphids and caterpillars? We quantified the transcript levels of two marker genes related to JA- and SA-signalling, i.e., *LIPOXYGENASE* (*LOX*) and *PATHOGENESIS-RELATED PROTEIN-1* (*PR-1*), respectively (Bell et al. 1995; Jirage et al. 2001), at different time points following inoculation by each of the three different chewing herbivore species and the piercing-sucking aphid when introduced alone, simultaneously, or sequentially on wild cabbage plants from populations.

## Materials and Methods

### Plants and Insects

Seeds of wild cabbage (*Brassica oleracea*) populations were collected in Dorset, U.K., at sites known as Kimmeridge (50°36′N, 2°07′W), Old Harry (50°38′N, 1°55′W), and Winspit (50°35′N, 2°02′W), hereafter called KIM, OH, and WIN, respectively. At each site, 20 plants were randomly selected for seed collection, and the seeds were pooled per site. Plants were grown from seeds in 1.5-L pots (1 plant per pot) containing potting soil (Lentse potgrond no. 4; Lent, The Netherlands) in a greenhouse (22 ± 3 °C, 50–70 % relative humidity [RH], light:dark regime [L:D] 16:8 h). Plants were placed in large trays (675 × 170 cm) that were automatically flooded with water and nutrients (NH4 1.2, K 7.2, Ca 4.0, Mg 1.82, NO3 12.4, SO4 3.32, P 1.0, Fe 35.0, Mn 8.0, Zn 5.0, B 20.0, Cu 0.5, Mo 0.5 in mmol/L) once every day for 20 min.

Except for *M. brassicae,* all other herbivore species (*B. brassicae*, *P. xylostella*, and *P. brassicae*) are specialist feeders on brassicaceous plant species, although *M. brassicae* is considered a pest on cabbage crops like the other three herbivore species. All insect cultures were maintained on Brussels sprouts (*B. oleracea* L. var. gemmifera cv. Cyrus) plants in a greenhouse or a climate room at 22 ± 2 °C, 60–70 % RH and 16:8 h L:D photoregime.

### General Treatment and Sampling Protocol

In a pilot experiment, we first determined the amount of damage inflicted in 24 h by each caterpillar species, and then adjusted the number of caterpillars per species to standardize the consumed area of leaf tissues. We used a transparent plastic sheet with a 1-mm^2^ grid to quantify the area of the consumed leaf tissue in mm^2^. Results of the pilot showed that three 2-d-old second instar (L2) *P. xylostella*, four 1-d-old L1 *M. brassicae*, and three neonate *P. brassicae* larvae, respectively, consume similar amounts of leaf tissue (*P. xylostella,* 53 ± 4.6; *M. brassicae,* 59 ± 6.0; *P. brassicae,* 49.9 ± 2.3 mm^2^) in 24 h. These numbers of caterpillars were used to inoculate the plants in the experiments described below. The initial inoculation density of *B. brassicae* was set at 8 adult aphids per plant.

Plants were exposed to herbivory treatments when they were 4 wk. old, and had 3–6 expanded leaves. Insects were introduced onto the first fully expanded leaf. To confine the insects to this leaf, the leaf petiole was wrapped with cotton wool. In each of the three experiments described below, one set of plants served as a control and was not exposed to herbivory, but was otherwise treated similarly. Plants of the three cabbage populations, exposed to different herbivory treatments, were placed randomly on the tables in a greenhouse. For gene-transcript quantification, per plant two leaf discs were punched with a cork-borer (diam 1.8 cm) from the herbivore-exposed leaves, and leaf discs were collected from three plants and pooled (=one replicate sample). Insects remained on the plants during the various exposure periods, and were only removed just before leaf sample collection. Twelve plants were prepared to obtain four replicate samples in total for each plant population, herbivore treatment, and time-point combination (see below). At each time point, an equal number of samples was collected from control plants, and a new cohort of control plants was used at each time point. Plants were sampled only once, i.e., distinct groups of plants were prepared for each time-treatment combination. Immediately after sample collection, samples were flash-frozen in liquid nitrogen and stored in a freezer at −80 °C until further processing for qRT-PCR.

### Experiment 1: Single Vs. Dual Infestation with *B. brassicae* Aphids and *P. xylostella* Caterpillars

Plants were inoculated with either *B. brassicae* (B) or *P. xylostella* (Px), or a combination of simultaneous *B. brassicae* and *P. xylostella* inoculation (Px + B). Samples for gene expression were collected at 6, 24, and 48 h after introduction of the herbivores as described in the previous section.

### Experiment 2: Effect of the Order of Arrival of *B. brassicae* Aphids and *P. xylostella* Caterpillars

Plants initially were inoculated with *B. brassicae* aphids or *P. xylostella* caterpillars, or left free of herbivores. The insects were allowed to feed and reproduce (aphids only) for 5 d. Following this incubation with the first herbivore, half of the plants that were exposed to each of the two herbivore treatments were co-infested with the other herbivore (coded BPx and PxB), whereas the remaining half of the plants were left as they were (B and Px). In addition, cohorts of plants that had not been exposed to herbivores previously, were inoculated with either *B. brassicae* or *P. xylostella* caterpillars (CB and CPx). Samples for gene expression were collected from all plants including controls (=without any herbivory) at 24 and 48 h after the second herbivore had been introduced.

### Experiment 3: Effect of Sequential Infestation with *B. brassicae* Aphids and Caterpillars of Different Herbivore Species

Sets of plants were or were not inoculated with *B. brassicae* aphids, and were incubated for 5 d, and then were infested with caterpillars of one of three different lepidopteran species, i.e., *P. xylostella*, *P. brassicae*, or *M. brassicae* (without aphids CPx, CPb, and CMb, and with aphids BPx, BPb, and BMb). Samples for gene expression were collected from all plants including controls (=without any herbivory) at 24 and 48 h after the second herbivore had been introduced.

### RNA Isolation and Real-Time Quantitative Reverse Transcription PCR (qRT-PCR)

Samples were kept frozen with liquid nitrogen, and ground to a fine powder with a mortar and pestle. RNA was isolated from homogenized material by using RNeasy Plant Mini Kit (Qiagen), and were treated with DNAsel (Invitrogen) following the manufacturer’s instructions. After isolation, the RNA concentration and purity were measured using a NanoDrop ND-100 (NanoDrop Technologies, Wilmington, DE, USA) spectrophotometer (all samples with OD 260 nm ⁄ 280 nm of 1.9–2.2 ratio). RNA integrity numbers (RIN) of randomly selected samples were confirmed by Bioanalyzer (Agilent 2100) with the Agilent RNA 6000 Nano Kit (Agilent Technologies, Waldbronn, Germany). The concentration of RNA obtained from the plant material was adjusted to 1 μg/μl, and subsequently was reverse-transcribed into cDNA with the iScript cDNA synthesis Kit (Bio-Rad). RNA samples were randomly selected for a negative control cDNA reaction by omitting the reverse transcriptase, to ensure that no samples were contaminated with genomic DNA. Quantitative Reverse-Transcriptase PCR (qRT-PCR) analysis was performed in an Mx3000P™ real-time PCR Detection system (Rotorgene). The qPCR amplification mix consisted of: 12.5 μl of SYBER Green Supermix (Bio-Rad), 5 μl cDNA, 5.5 μl DEPC-treated water, and 10 pmol of each primer in 1 μl each (see Table 1, Wageningen, The Netherlands) adding up to a total volume of 25 μl. The primers of the two tested genes were designed based on those used in the study by Zheng et al. (2007) and Poelman et al. (2011). The amplification efficiency of primers was determined by generating standard curves using a 10-fold dilution of the randomly selected samples per treatment and per cabbage population. Each dilution was assayed in triplicate. The amplification efficiency was between 90 and 100 % for all primer pairs tested on the three cabbage populations. For each cDNA sample, qPCR amplification reactions were performed in duplicate. The following PCR program was used for all amplification reactions: an initial denaturation step of 3 min at 95 °C, followed by 40 cycles of 15 s at 95 °C, 45 s at 59 °C. At the end of each run, melting curve analysis was performed to verify that only a single gene transcript had been amplified. Relative gene transcript levels were calculated by normalizing transcript levels to the threshold cycle (Ct) values of the reference gene GAPDH using the 2^-ΔΔCt^ method (Livak and Schmittgen 2001). The Ct values of the reference gene GAPDH were consistent across treatments.

**Table 1:** Primer sequences used for amplifying *Gapdh*, *Pr-1*, And *Lox* genes of *Brassica oleracea*

Gene name	Forward primer	Reverse primer
*BoGAPDH*	5′-AGAGCCGCTTCCTTCAACATCATT-3’	5′-TGGGCACACGGAAGGACATACC-3’
*BoLOX*	5′-AAGGCATCGAGCTTCCCAA-3’	5′-TTGCTTTTCAACGGCCACTC-3’
*BoPR-1*	5′-GTCAACGAGAAGGCTAACTATAACTACG-3’	5′-TTACACCTTGCTTTGCCACATCC-3’

### Statistics

The response variables, relative transcript levels of *LOX* and *PR-1,* were log-transformed to meet assumptions of normality and homoscedasticity. Data were analyzed using General Linear Model analysis of variance in Genstat (17th edition, VSN International, Hemel Hempstead, UK). In experiments 1 and 2, plant population, herbivore treatment and time points were entered as fixed factors in the statistical model. The data of experiment 2 were split into two sets: data of gene transcript levels of control plants and those exposed to aphid infestation alone (C, CB, and B) were analyzed separately, to confirm the effect of aphid infestation in experiment 1; data of gene transcript levels of plants with caterpillar (*P. xylostella*) infestation alone and in combination with aphid feeding (CPx, Px, BPx, and PxB) were analyzed to investigate the effect of temporal order of infestation. In experiment 3, we investigated whether transcript levels of the two genes were similarly affected by the infestation of different caterpillar species, both in the presence and absence of aphid feeding. In addition to population and time points, caterpillar species and presence / absence of aphids were entered as fixed terms in the statistical model. When terms in the GLM were significant, pairwise differences among factor levels were determined using Tukey-Kramer-corrected LSD tests.

## Results

### Experiment 1: Single Vs. Dual Infestation with *B. brassicae* Aphids and *P. xylostella* Caterpillars - *LOX* Expression

**Fig. 1:**
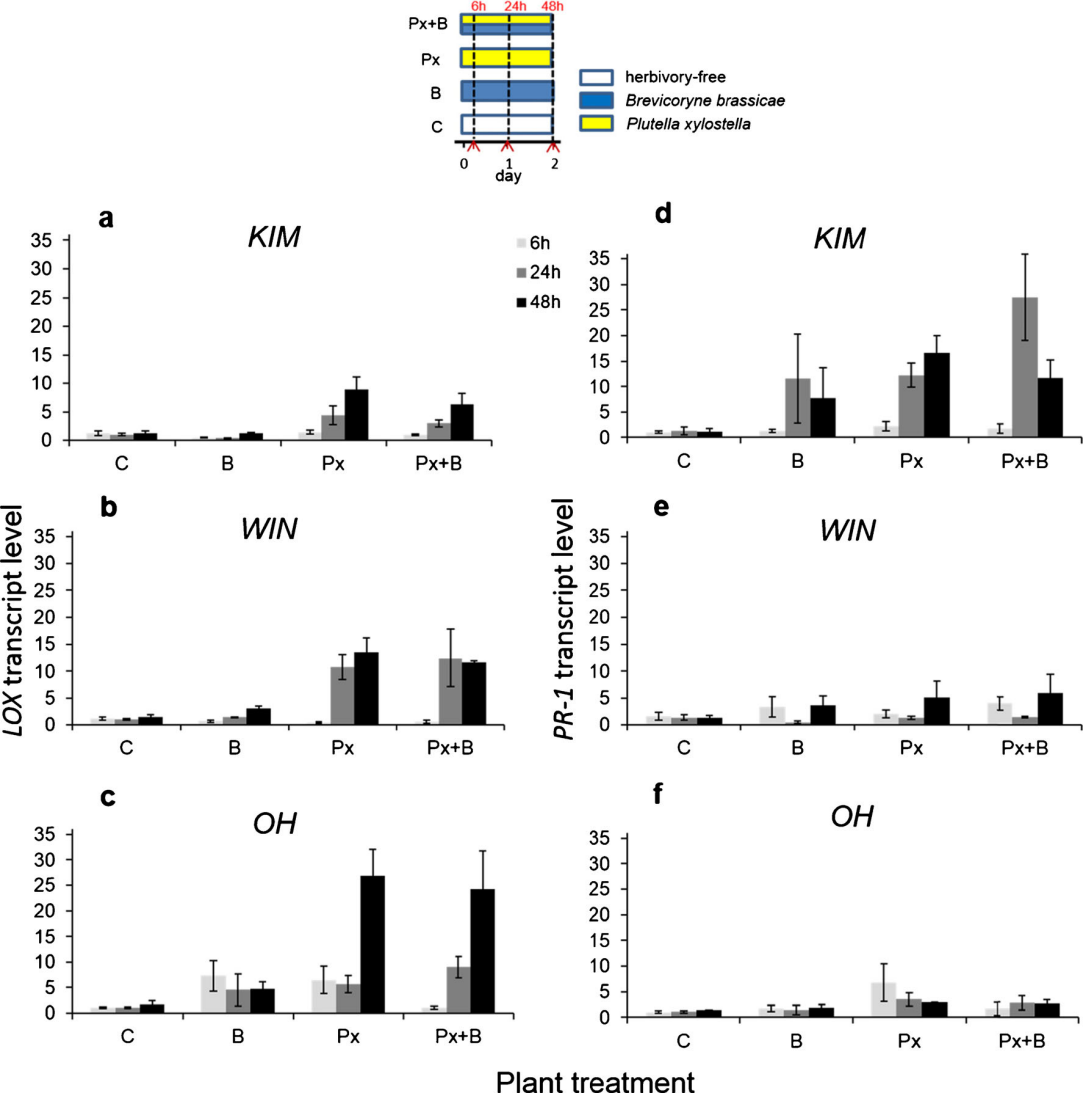
Quantitative RT-PCR analysis of transcript levels of the jasmonic acid (JA)-responsive defense marker gene *LOX* (panels **a**-**c**) and the salicylic acid (SA)-responsive defence marker gene *PR-1* (panels **d**-**f**) in leaves of plants from three different wild *Brassica oleracea* populations (KIM [**a;d**]; WIN [**b;e**]; OH [**c;f**]) at 6, 24, and 48 h after infestation with *Plutella xylostella* caterpillars (Px); *Brevicoryne brassicae* aphids (B); both *P. xylostella* and *B. brassica* simultaneously (Px + B), or without any herbivory (C). Transcript levels of genes are shown as fold changes in mean relative expression compared to those of control plants (**C**). Bars present means ± SE (*N* = 4). The temporal sequence of plant inoculation with different insect herbivore species is given in a schematic overview above the bar graphs. Sampling time points for gene transcript analysis are indicated by the red coloured arrows on the X-axis

There was a significant effect of herbivore treatment, time point, and population on the transcript levels of *LOX* (Table 2, Fig. 1a-c). Feeding by *P. xylostella* caterpillars alone or in combination with *B. brassicae* aphids (Fig. 1a) similarly up-regulated the expression of *LOX* (Px vs. Px + B, *P* = 0.32), whereas transcript levels of *LOX* were similar in the controls and in plants exposed to *B. brassicae* alone (C vs. B, *P* = 0.71). The marginally significant interaction between herbivore treatment and plant population further indicated that the extent to which *LOX* was transcribed was somewhat plant-population specific. The relative expression of *LOX* in caterpillar-exposed plants increased with time, and the temporal dynamics of this gene also differed among the populations (Table 2). For example, in KIM plants, *LOX* transcripts were significantly different only in samples taken at 6 and 48 h following herbivore introduction (KIM-6 vs. KIM-48, *P* = 0.02), whereas in WIN plants transcript levels differed at both 24 and 48 h from those at 6 h (*P* < 0.001 both comparisons). In OH plants, the patterns were similar to those in KIM plants, but they were not statistically significant due to the high levels of variation (6 h vs. 48 h *P* = 0.10).

**Table 2:** GLM analysis results of the main effects of wild cabbage (*Brassica oleracea*) plant population, herbivore treatment, time point and their interaction terms on the transcript level of *lox* and *pr-1* in experiment 1 ^a^

Experiment 1					
Tested gene	Factor	*N.d.f.*	*D.d.f.*	*F* statistic	*P* value
*LOX*	Plant population (1)	2	107	12.57	**< 0.001**
	Treatment (2)	3	107	15.16	**< 0.001**
	Time point (3)	2	107	36.08	**< 0.001**
	Interaction 1*2	6	107	1.99	0.074
	Interaction 1*3	4	107	6.78	**< 0.001**
	Interaction 2*3	6	107	8.41	**< 0.001**
	Interaction 1*2*3	12	107	1.10	0.368
*PR-1*	Factor	*N.d.f.*	*D.d.f.*	*F* statistic	*P* value
	Plant population (1)	2	105	7.67	**< 0.001**
	Treatment (2)	3	105	7.07	**< 0.001**
	Time point (3)	2	105	3.07	0.051
	Interaction 1*2	6	105	1.40	0.223
	Interaction 1*3	4	105	4.99	**0.001**
	Interaction 2*3	6	105	1.40	0.222
	Interaction 1*2*3	12	105	1.15	0.331

*P*-values in bold denote significant effects

^a^ In the statistical model, plant population had three levels (KIM, WIN, OH), treatment had four levels (see Fig. 1) and time point had three levels (6, 24, and 48 h)

### *PR-1* Expression

The transcript levels of *PR-1,* a SA-responsive marker gene, were affected by herbivore treatment and plant population, and the effect of time point was population specific (Table 2, Fig. 1d-f). The expression of this gene was up-regulated only in KIM plants (KIM vs. OH, *P* = 0.005, KIM vs. WIN, *P* = 0.01; WIN vs. OH, *P* = 0.97), and the transcript levels increased only in response to feeding by *P. xylostella* alone or in combination with aphids (B vs. C, *P* = 0.77, Px vs. Px + B, *P* = 0.92, all other pair-wise comparisons *P* < 0.05). In KIM plants, *PR-1* transcript levels were higher in tissue sampled at 24 h than in those sampled at 6 h following infestation (KIM-6 vs. KIM-24, *P* = 0.007; all other within population-time point comparisons *P* > 0.05).

### Experiment 2: Effect of the Order of Arrival of *B. brassicae* Aphids and *P. xylostella* Caterpillars

**Fig. 2:**
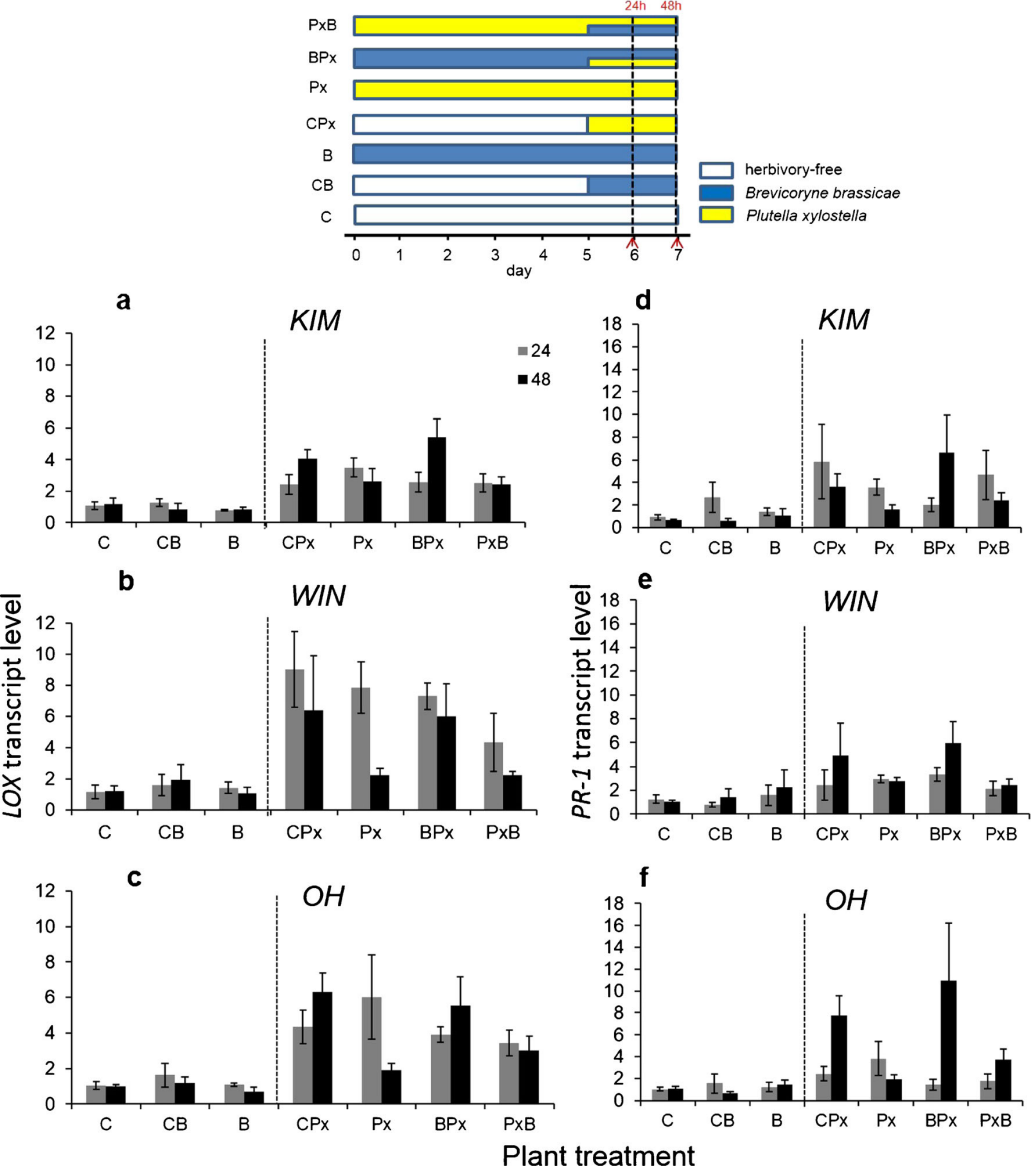
Quantitative RT-PCR analysis of transcript levels of the jasmonic acid (JA)-responsive defense marker gene *LOX* (panels a-c) and a salicylic acid (SA)-responsive defence marker gene *PR-1* (panels d-f) in leaves of plants from three wild *Brassica oleracea* populations (KIM [a;d]; WIN [b;e]; OH [c;f]). Plants were infested with *Plutella xylostella* or *Brevicoryne brassicae* either at d 0 (Px and B) or d 5 (CPx and CB), or they were dually infested with *P. xylostella* at d 0 and with *B. brassicae* at d 5 (PxB), or with *B. brassicae* at d 0 and with *P. xylostella* at d 5 (BPx). Gene expression was measured 24 and 48 h following treatment with the second herbivore. Gene transcript levels are shown as fold changes in mean relative expression compared to those in herbivore free control plants (C). Bars present means ± SE (*N* = 4). The temporal sequence of plant inoculation with different insect herbivore species is given in a schematic overview above the bar graphs. Sampling time points for gene transcript analysis are indicated by the red coloured arrows on the X-axis

In a first analysis including data from control plants (C) and plants infested with aphids (CB and B) alone (for treatment coding see Fig. 2), we confirmed the results of experiment 1. The transcript levels of both *LOX* and *PR-1* were similar in plants infested by *B. brassicae* alone for a short period, i.e., 1 or 2 days (CB), or an extended period, i.e., 6 or 7 days (B) compared to control plants (C), irrespective of the plant population (Table 3a; Fig. 2). In a second analysis, we investigated the effect of the temporal infestation order of *P. xylostella* caterpillars and *B. brassicae* aphids on gene transcript levels.

**Table 3:** GLM analysis results of the main effects of plant population, herbivore treatments, time point and their interaction terms on the transcript level of *lox* and *pr-1* in experiment 2 ^a^

Experiment 2(a) Treatment of C, CB, B					
Tested gene	Factor	*N.d.f.*	*D.d.f*	*F* statistic	*P* value
*LOX*	Plant population (1)	2	53	0.30	0.739
	Treatment (2)	2	53	0.56	0.572
	Time point (3)	1	53	1.92	0.171
	Interaction 1*2	4	53	0.14	0.968
	Interaction 1*3	2	53	0.04	0.956
	Interaction 2*3	2	53	0.53	0.593
	Interaction 1*2*3	4	53	0.29	0.883
*PR-1*	Factor	*N.d.f.*	*D.d.f*	*F* statistic	*P* value
	Plant population (1)	2	53	0.09	0.918
	Treatment (2)	2	53	0.75	0.479
	Time point (3)	1	53	0.71	0.403
	Interaction 1*2	4	53	0.04	0.996
	Interaction 1*3	2	53	1.54	0.224
	Interaction 2*3	2	53	0.76	0.473
	Interaction 1*2*3	4	53	0.60	0.664
Experiment 2(b) Treatment of CPx, Px, BPx,PxB					
Tested gene	Factor	*N.d.f.*	*D.d.f*	*F* statistic	*P* value
*LOX*	Plant population (1)	2	72	4.33	**0.017**
	Treatment (2)	3	72	4.57	**0.005**
	Time point (3)	1	72	3.31	0.073
	Interaction 1*2	6	72	0.45	0.846
	Interaction 1*3	2	72	6.02	**0.004**
	Interaction 2*3	3	72	4.35	**0.007**
	Interaction 1*2*3	6	72	0.45	0.845
*PR-1*	Factor	*N.d.f.*	*D.d.f*	*F* statistic	*P* value
	Plant population (1)	2	78	0.07	0.929
	Treatment (2)	3	78	0.74	0.532
	Time point (3)	1	78	5.17	**0.026**
	Interaction 1*2	6	78	0.57	0.751
	Interaction 1*3	2	78	1.71	0.187
	Interaction 2*3	3	78	3.99	**0.011**
	Interaction 1*2*3	6	78	0.52	0.793

*P*-values in bold denote significant effects

^a^ In the statistical model, plant population had three levels (KIM, WIN, OH), treatment had three levels for experiment 2(a), four levels for experiment 2(b) (see Fig. 2) and time point had two levels (24 and 48 h)

### *LOX* Expression

Herbivore treatment and plant population had a significant effect on the expression of *LOX* (Table 3b, Fig. 2a-c). Overall, the presence of *B. brassicae* had relatively little effect on the expression of *LOX*, regardless of the order of arrival (CPx vs. BPx, *P* = 0.72, Px vs. PxB, *P* = 0.99, Fig. 2a-c). *LOX* transcription differed only between plants that were infested with caterpillars first and aphids second and between plants that were infested with caterpillars late, irrespective of whether there were also aphids on the plant (PxB vs. CPx, *P* = 0.02; PxB vs. BPx, *P* = 0.01). However, there also was a significant interaction between the time point of sampling and treatment. At both time points, *LOX* transcript levels were equally high in plants that were infested with caterpillars late, irrespective of the presence of aphids (CPx-24 h vs. CPx-4 h and BPx-24 h vs. BPx-48 h, *P* > 0.95). In plants infested with caterpillars early and no aphids, *LOX* transcript levels were significantly lower at 48 than at 24 h (Px-24 h vs. Px-48 h, *P* = 0.006), whereas in plants infested with caterpillars early and aphids late (PxB), transcription levels were equally low at both time points (*P* > 0.05). The late infestation of *B. brassicae* and the extended period of caterpillar feeding tended to suppress the transcript level of *LOX* (PxB-48 h vs. Px-48 h). *LOX* transcripts were higher in WIN than in KIM plants, whereas levels of this gene in OH plants did not differ from those in plants from the other two populations (Fig. 2a-c, WIN vs. KIM, *P* = 0.01, OH vs. KIM, *P* = 0.17, and OH vs WIN, *P* = 0.53). The effect of time point on *LOX* expression differed among plant populations. The up-regulation of *LOX* was fastest in WIN (levels were higher at 24 h than at 48 h, *P* = 0.006), whereas for the other two populations, there was no difference between transcript levels at 24 and 48 h (KIM-24 vs. KIM-48, *P* = 0.84, OH-24 vs. OH-48, *P* = 0.99).

### *PR-1* Expression

The effect of herbivore treatment on *PR-1* transcription depended on the time of sampling (Table 3b, Fig. 2d-f). *PR-1* transcript levels did not differ among the plant populations (Table 3b). In the treatments where caterpillars were introduced late, i.e., CPx and BPx, *PR-1* transcript levels were higher at 48 than at 24 h, but this was significant only in the treatment where aphids were introduced first and caterpillars second (BPx-24 vs. BPx-48, *P* = 0.05).

### Experiment 3: Effect of Dual Infestation with *B. brassicae* Aphids and Caterpillars of Different Herbivore Species

#### *LOX* Expression

**Fig. 3:**
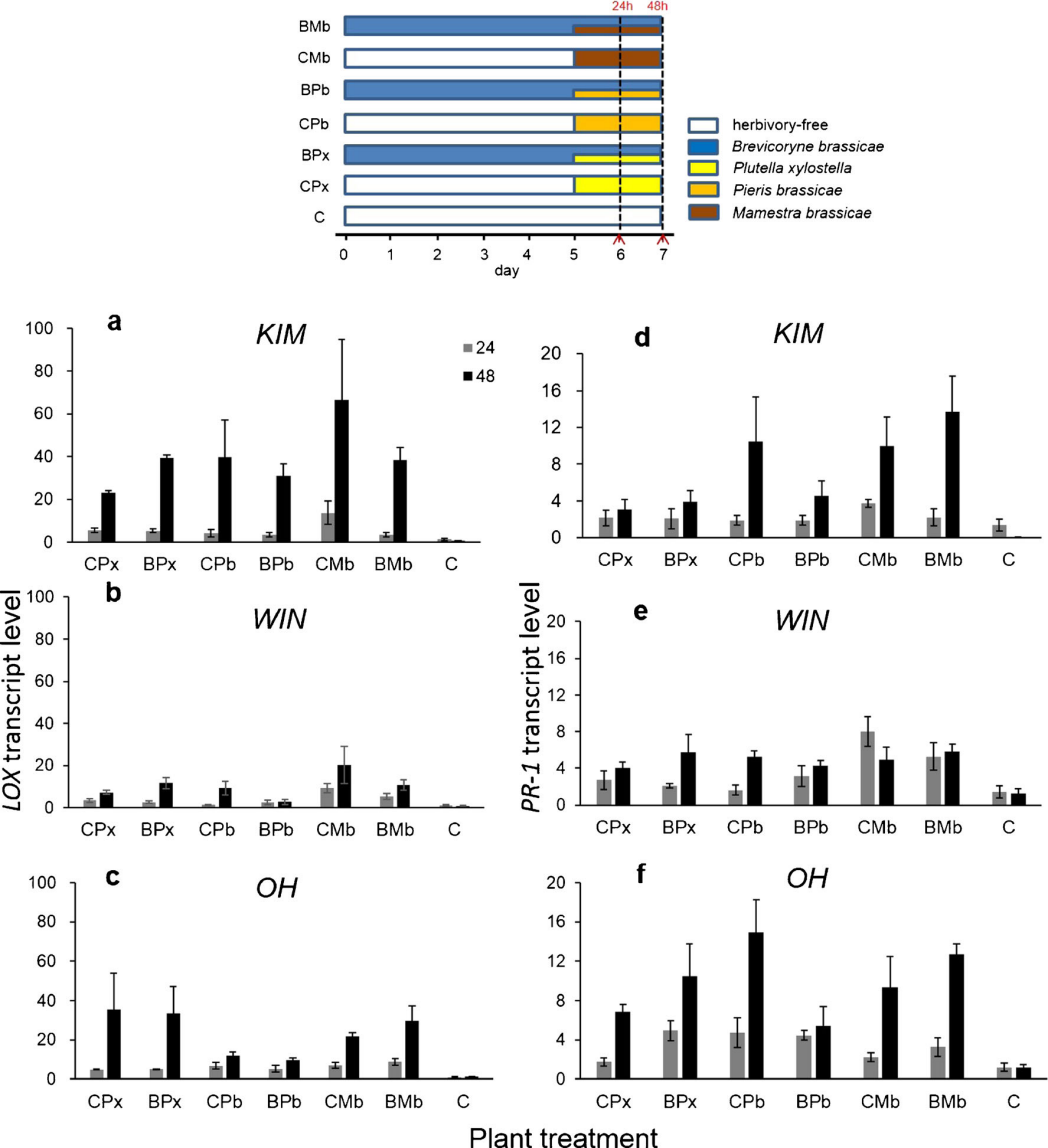
Quantitative RT-PCR analysis of transcript levels of the jasmonic acid (JA)-responsive defense marker gene *LOX* (panels **a**-**c**) and a salicylic acid (SA)-responsive defense marker gene *PR-1* (panels **d**-**f**) in leaves of wild *Brassica oleracea* populations (KIM [**a;d**]; WIN [**b;e**]; OH [**c;f**]). Plants were infested with caterpillars of one of three lepidopteran species *Plutella xylostella* (CPx)*, Pieris brassicae* (CPb), or *Mamestra brassicae* (CMb) on d 5, or they were dually infested with *Brevicoryne brassicae* at d 0 and caterpillars on d 5 (BPx, BPb, and BMb, respectively). Gene expression was measured 24 and 48 h following treatment with the second herbivore. Gene transcript levels are shown as fold changes in mean relative expression compared to those in herbivore free control plants (C). Bars present means ± SE (*N* = 4). The temporal sequence of plant inoculation with different insect herbivore species is given in a schematic overview above the bar graphs. Sampling time points for gene transcript analysis are indicated by the red colored arrows on the X-axis

The extent to which *LOX* was up-regulated was affected by caterpillar species, plant population, and the time of sampling, while it was not affected by the presence or absence of feeding aphids (Table 4; Fig. 3a-c). *LOX* transcript levels were highest in plants infested with *M. brassicae*, intermediate in plants infested with *P. xylostella*, and lowest in plants infested with *P. brassicae* (Fig. 3a-c; Mb vs. Pb, *P* < 0.001, Mb vs. Px, *P* = 0.03, Px vs. Pb, *P* < 0.001). Overall, transcript levels of *LOX* were higher in KIM and OH plants than in WIN plants (KIM vs. OH, *P* = 0.18, WIN vs. KIM, and WIN vs. OH, *P* < 0.001), and they were higher at 48 h than at 24 h after initiation of caterpillar feeding. However, the extent to which transcript levels increased with time depended on the population; whereas transcript levels were similar at 24 h following the introduction of the herbivores (all comparisons *P* > 0.05), transcript levels at 48 h were highest in KIM, intermediate in OH, and lowest in WIN (KIM vs. OH, *P* = 0.005, KIM vs. WIN and OH vs. WIN, *P* < 0.001).

**Table 4:** GLM analysis results of the main effects of plant population, herbivore treatment, time point and their interaction terms on the transcript level of *lox* and *pr-1* in experiment 3 ^a^

Experiment 3					
Tested gene	Factor	*N.d.f.*	*D.d.f*	*F* statistic	*P* value
*LOX*	Plant population (1)	2	108	37.69	**< 0.001**
	Caterpillar infestation (2)	2	108	20.98	**< 0.001**
	*B. brassicae* infestation (3)	1	108	2.82	0.096
	Time point (4)	1	108	190.11	**< 0.001**
	Interaction 1*2	4	108	2.17	0.077
	Interaction 1*3	2	108	0.90	0.41
	Interaction 2*3	2	108	2.50	0.087
	Interaction 1*4	2	108	12.39	**< 0.001**
	Interaction 2*4	2	108	0.81	0.45
	Interaction 3*4	1	108	0.32	0.57
	Interaction 1*2*3	4	108	1.66	0.16
	Interaction 1*2*4	4	108	2.00	0.10
	Interaction 1*3*4	2	108	1.47	0.23
	Interaction 2*3*4	2	108	2.65	0.075
	Interaction 1*2*3*4	4	108	1.25	0.30
*PR-1*	Factor	*N.d.f.*	*D.d.f*	*F* statistic	*P* value
	Plant population (1)	2	108	6.83	**0.002**
	Caterpillar infestation (2)	2	108	6.30	**0.003**
	*B. brassicae* infestation (3)	1	108	0.07	0.799
	Time point (4)	1	108	59.87	**< 0.001**
	Interaction 1*2	4	108	2.43	0.052
	Interaction 1*3	2	108	1.35	0.263
	Interaction 2*3	2	108	1.78	0.174
	Interaction 1*4	2	108	3.42	**0.036**
	Interaction 2*4	2	108	0.05	0.948
	Interaction 3*4	1	108	0.11	0.742
	Interaction 1*2*3	4	108	1.40	0.238
	Interaction 1*2*4	4	108	2.66	**0.036**
	Interaction 1*3*4	2	108	1.14	0.324
	Interaction 2*3*4	2	108	5.01	**0.008**
	Interaction 1*2*3*4	4	108	0.10	0.983

*P*-values in bold denote significant effects

^a^In the statistical model, plant population had three levels (KIM, WIN, OH), treatment had seven levels (see Fig. 3) and time point had two levels (24 and 48 h)

#### *PR-1* Expression

The results for transcript levels of *PR-1* in response to feeding by different caterpillar species in the presence or absence of aphids were more idiosyncratic; two of the four three-way interactions were significant (Table 4, Fig. 3d-f). Overall, *PR-1* transcripts increased more in response to *M. brassicae* than to *P. xylostella* feeding (*P* = 0.002), whereas transcription of this gene was similar in response to *P. brassicae* feeding and feeding by the other two caterpillar species (Pb vs. Px and Pb vs. Mb, *P* > 0.05). Early aphid infestation did not affect transcript levels of *PR-1* in response to *P. xylostella* and *M. brassicae* feeding, whereas it had a tendency to decrease *PR-1* transcript levels in plants infested with *P. brassicae* larvae for 48 h; however, this was not statistically significant. Overall transcript levels of *PR-1* in OH plants were higher than in the other two populations (OH vs. KIM, *P* < 0.001, OH vs. WIN, *P* = 0.04, KIM vs. WIN, *P* = 0.46). Transcript levels of *PR-1* were higher at 48 than at 24 h following the introduction of the caterpillars, but the extent of this increase was plant-population dependent.

## Discussion

Both marker genes, *LOX* and *PR-1,* were up-regulated in response to a single *P. xylostella* infestation in all three cabbage populations, whereas a single infestation by *B. brassicae* aphids did not affect transcript levels of either of these two genes. In addition, dual infestation with aphids and *P. xylostella* caterpillars, simultaneously or separated in time (regardless of the order of infestation) had little or no effect on transcription levels of *LOX* and *PR-1* compared to the transcript levels in treatments with individual herbivores. Caterpillar species differentially affected up-regulation of the two marker genes. As was found for *P. xylostella*, aphid presence did not interfere with transcription of *LOX* and *PR-1* in response to feeding by *P. brassicae* or *M. brassicae* caterpillars. The main effects were consistent across the three cabbage populations, although there were population-related differences in the temporal dynamics of *LOX* and *PR-1* transcription, in the response to the three caterpillar species, and also in the extent to which plants of the three populations up-regulated gene expression in response to the various herbivore treatments.

Based on negative SA-JA crosstalk, we hypothesized that aphid infestation would lead to higher transcript levels of SA-responsive genes and would suppress the transcription of JA-responsive genes in response to caterpillar attack. The temporal order of herbivore attack can further influence the timing and intensity of plant defense responses to aphid and caterpillar feeding and their interaction (Erb et al. 2011; Stam et al. 2014). In contrast to our hypothesis, the results of the present study show no effects of aphid infestation on the transcript levels of a JA- and a SA-responsive gene when plants were challenged by both *B. brassicae* and different caterpillar species, irrespective of the temporal sequence of aphid and caterpillar attack. The lack of interference with transcription of JA- or SA-responsive genes by aphid infestation on the *B. oleracea* plants may be attributed to: 1) a lack of effects of aphid infestation on SA production, as was also reported by Ali and Agrawal (2014) for *Asclepias tuberosa*. Low transcript levels of *PR-1* in the present study may imply overall low activation of SA signalling in *B. oleracea* plants in response to aphid infestation. 2) Absence of negative crosstalk between SA and JA in *B. oleracea*. A review by Thaler et al. (2012) reported the absence of SA-JA antagonism in several plant species, e.g., *Zea mays* (Poaceae), *Asclepias exaltata* (Apocynaceae), and *Picea abies* (Pinaceae), suggesting that this phenomenon is not ubiquitous across taxa even when they are in the same family like *A. thaliana* and *B. oleracea*. 3) The temporal kinetics of JA- and SA-mediated defense induction and concomitant gene expression in wild cabbage may differ from those reported for the model plant *A. thaliana.* 4) In wild *B. oleracea*, genes other than *LOX* and *PR-1* are involved in JA-SA antagonism. Alternatively, the selected genes may not be involved in negative JA-SA cross talk in wild *B. oleracea.* 5) The duration of the time interval of aphid feeding (5 days) before the caterpillars were introduced onto the plants was not long enough to have a measurable effect on transcription levels of the genes examined. In some other systems, aphid feeding has been shown to take up to a week to elicit chemical defences (Appel et al. 2014; Mewis et al. 2005, 2006).

Aphid (*B. brassicae*) feeding alone had no effect on the transcript levels of either *PR-1*, a gene supposedly responsive to aphid feeding (De Vos et al. 2005) or *LOX*. This is consistent with data by Moran and Thompson (2001) who reported that *B. brassicae* did not induce responses associated with JA- or SA-related metabolic processes in *A. thaliana* plants. Due to the stealthy feeding style of aphids and the minimal wounding they cause to plants, induction of SA in response to aphid herbivory occurs very locally, i.e., only at the site of aphid feeding, and, therefore, transcript levels of *PR-1* may be low and difficult to detect (De Vos et al. 2005). Previous studies have reported that the saliva of aphids is rich in elicitors (Hogenhout and Bos 2011; Walling 2000). Both SA- and JA-responsive genes reacted to feeding by *B. brassicae* and *M. persicae* in Arabidopsis (Kusnierczyk et al. 2008, 2011; Moran and Thompson 2001). However, the aphid-induced responses of plants also can be aphid species-specific. For instance, *M. persicae* feeding induced SA-responsive genes in *A. thaliana*, whereas *B. brassicae* did not (Appel et al. 2014). *Brevicoryne brassicae* may have evolved to avoid inducing defensive responses in wild cabbage plants that are often attacked by this aphid in its natural habitat (Newton et al. 2009a), or it may manipulate the plant defense for its own benefit (Howe et al. 2007; Walling 2008). This also has been reported for a population of the herbivorous spider mite *Tetranychus urticae* that does not induce JA-regulated defenses in tomato that negatively affect spider mite performance (Kant et al. 2008).

Feeding by *P. xylostella* caterpillars significantly up-regulated gene expression not only of *LOX*, but also of *PR-1*, irrespective of whether the caterpillars were feeding alone or together with aphids. Studies by Kroes et al. (2015) and Ehlting et al. (2008) showed similar results, confirming that *P. xylostella* induces the expression of both SA- and JA-responsive genes in Arabidopsis. Glucose oxidase, present in the saliva of *Helicoverpa zea* caterpillars, induces SA signalling, which leads to inhibition of JA signalling and eventually prevents the induction of nicotine in *Nicotiana tabacum* plants (Musser et al. 2002, 2005). In our study, not only infestation by *P. xylostella*, but also by two other lepidopteran species, *P. brassicae* and *M. brassicae,* consistently up-regulated the expression of both *LOX* and *PR-1* genes on all three cabbage populations, regardless of the differences in dietary specialization and salivary elicitors (Felton 2008). In previous studies, it has been shown that both *P. xylostella* and *P. brassicae*, but not *M. brassicae* gained fitness benefits by feeding on wild and cultivated cabbage plants co-infested with *B. brassicae* aphids (Li et al. 2014; Soler et al. 2012). However, as we did not record an effect of aphid infestation on the transcription of the JA-responsive gene *LOX*, it remains to be investigated whether the enhanced performance of *P. xylostella* and *P. brassicae* (Li et al. 2014; Soler et al. 2012) results from attenuation of JA-mediated defenses. Alternatively, negative interference between JA and SA may affect genes other than *LOX* and *PR-1* in wild *B. oleracea.*

The extent to which *LOX* and *PR-1* were up-regulated differed among plants infested by different species of caterpillars: infestation with *M. brassicae* up-regulated both genes more compared to infestation by *P. brassicae* or *P. xylostella*. Herbivores with a similar feeding mode tend to induce more similar transcriptome responses in *A. thaliana* plants than herbivores with a different feeding mode (Appel et al. 2014; Bidart-Bouzat and Kliebenstein 2011; Ehlting et al. 2008). However, for transcriptional responses induced by the chewing herbivores *P. xylostella* or *P. rapae*, only 32 to 40 % of the genes were elicited commonly (Ehlting et al. 2008). Thus, induction of signal transduction components in plants may differ among herbivore species, even when the attacking herbivore species are from the same feeding guild (Bidart-Bouzat and Kliebenstein 2011; Diezel et al. 2009; Mewis et al. 2006). Although the chewing herbivores in this study are all members of the Lepidoptera, they differ in their feeding behavior. First and second instar *P. xylostella* larvae usually mine the leaf spongy mesophyll tissues, while later instars feed on the abaxial surface of leaves often leaving the upper epidermis intact (Sarfraz et al. 2005). *Pieris brassicae* larvae chew the leaf tissues gregariously, and initially cause a single damage site on the leaf. *Mamestra brassicae* larvae disperse immediately after egg hatching and then feed solitarily causing scattered sites of feeding damage on the leaves. It remains unknown to what extent the different feeding patterns of these caterpillars contribute to the differences in induced plant transcriptional responses.

At the plant population level, we found differences in the overall transcriptional responses of plants to the various treatments and also in the temporal dynamics of these responses. These population-related differences were not consistent, however, among the various experiments. This suggests that variation in conditions that could not be controlled, either related to the greenhouse environment or the plants themselves, may have resulted in population-specific variation in the response to the various treatments. These results reveal that the expression of genes involved in JA and SA defense signalling can be quite subtle and linking gene expression to responses occurring at a higher level of biological organization should be done with caution. Nevertheless, also at the population level, there is no evidence to support JA-SA antagonism based on transcript levels of the two marker genes.

The non-interactive effects of aphid and caterpillar infestation on the transcription levels of JA- and SA- responsive marker genes in the wild cabbage populations, regardless of the temporal sequence of both types of herbivory, implies that JA-SA antagonism may not occur ubiquitously in all plant taxa. In this study, we examined only two genes that are considered representative for the two signalling pathways. Future studies should aim to investigate genes with different functions, and genes that are transcribed along the sequence of molecular events that are activated in response to herbivory. The interaction between the JA and SA signalling pathways is likely to be far more complex involving various genes of which some interact antagonistically and others do not.
